# Different aspects in explaining how mutations could affect the binding mechanism of receptor binding domain of SARS-CoV-2 spike protein in interaction with ACE2

**DOI:** 10.1371/journal.pone.0291210

**Published:** 2023-09-08

**Authors:** Farzaneh Jafary, Farzane Abasi Joozdani, Kiana Shahzamani, Sepideh Jafari, Hossein Mirhendi, Mohamad Reza Ganjalikhany

**Affiliations:** 1 Core Research Facilities (CRF), Isfahan University of Medical Science, Isfahan, Iran; 2 Isfahan Endocrine and Metabolism Research Center, Isfahan University of Medical Sciences, Isfahan, Iran; 3 Department of Biophysics, Faculty of Biological Science, Tarbiat Modares University, Tehran, Iran; 4 Hepatitis Research Center, School of Medicine, Lorestan University of Medical Sciences, Khorramabad, Iran; 5 Centre of Molecular and Macromolecular Studies, Polish Academy of Sciences, Łódź, Poland; 6 Department of Medical Parasitology and Mycology, School of Public Health, Tehran University of Medical Sciences, Tehran, Iran; 7 Department of Cell and Molecular Biology & Microbiology, Faculty of Biological Science and Technology, University of Isfahan, Isfahan, Iran; Gauhati University, INDIA

## Abstract

During replication, some mutations occur in SARS-CoV-2, the causal agent of COVID-19, leading to the emergence of different variants of the virus. The mutations that accrue in different variants of the virus, influence the virus’ ability to bind to human cell receptors and ability to evade the human immune system, the rate of viral transmission, and effectiveness of vaccines. Some of these mutations occur in the receptor binding domain (RBD) of the spike protein that may change the affinity of the virus for the ACE2 receptor. In this study, several *in silico* techniques, such as MD and SMD simulations, were used to perform comparative studies to deeply understand the effect of mutation on structural and functional details of the interaction of the spike glycoprotein of SARS-CoV-2, with the ACE2 receptor. According to our results, the mutation in the RBD associated with the Omicron variant increase binding affinity of the virus to ACE2 when compared to wild type and Delta variants. We also observed that the flexibility of the spike protein of the Omicron variant was lower than in comparison to other variants. In summary, different mutations in variants of the virus can have an effect on the binding mechanism of the receptor binding domain of the virus with ACE2.

## Introduction

Many variants of severe acute respiratory syndrome coronavirus 2 (SARS-CoV-2), the causal agent of coronavirus disease 2019 (COVID-19), have emerged. Increased viral replication increases the likelihood of the creation of new SARS-CoV-2 mutations. It is believed that some variants are of particular importance due to their potential for increased transmissibility [1], higher virulence, and/or reduced effectiveness of vaccines [2, 3]. These variants contribute to the continuation of the COVID-19 pandemic. The term “variant of concern” (VOC) for SARS-CoV-2 refers to viral variants that have mutations in their spike protein receptor-binding domain (RBD), which improve the binding affinity of RBD with thehACE2 complex causing the faster spread of the virus in populations [4]. The World Health Organization (WHO) has considered Alpha, Beta, Gamma, Delta, and Omicron [5–7] as variants of concern.

The Delta variant (B.1.617.2) was initially discovered in India in late 2020 and then spread to over 163 countries by August 24, 2021. In June 2021, the WHO stated that Delta was going to be the most prevalent strain in the world [8]. Therefore, the Delta variant was changed from a variant of interest (VOI) to VOC. According to the present evidence, the Delta VOC was 40%–60% more transmissible than the Alpha (B.1.1.7) VOC and is probably associated with an increased risk of hospitalization. The Delta VOC mostly threatened those who were unvaccinated or partially vaccinated [9].

Another variant identified as a VOC was B.1.1.529, commonly known as Omicron. Following the discovery of Omicron, the Technical Advisory Group on SARS-CoV-2 Virus Evolution (TAG-VE) announced that this variant consisted of several mutations that might impact viral spread or the severity of the clinical symptoms of COVID-19. To date, 30 mutations, 15 of which occur in the RBD, as well as 3 small deletions and 1 minor insertion, have been identified as variants of the spike protein [6, 7]. These mutations alter the behavior of the virus, such as its pathogenicity and transmission. Therefore, the analysis of different variants will help us to discover the role of mutations in the pathogenesis of COVID-19 and how these mutations may impact the faster spread of the virus.

In our previous study, we tried to discover critical residues that participate in the interaction of SARS-CoV-2 with ACE2 [10]. We showed that mutations are able to alter the affinity of the virus for the ACE2 receptor. Wu et al. demonstrated that the Omicron variant exhibited a weaker binding affinity for the ACE2 receptor compared to the Delta variant [11]. In another study, Kumar and colleagues reported that the Omicron variant had a significant number of mutations in the RBD. These mutations in the Omicron variant have a greater affinity for human ACE2 than the Delta version, suggesting a higher risk of viral transmission [12]. Because there are conflicting reports about the effect of mutations on affinity of the different variant of virus for the ACE2 receptor, in the current study, we used MMPBSA and also SMD to discover the role of mutation on binding affinity of SARS‐CoV‐2, Delta, and Omicron variants with ACE2. We also try to discover more details about the binding mechanism between the receptor binding domain of the SARS-CoV-2 spike protein with ACE2 in tree variants.

## Results

### Analysis of mutations between SARS-CoV-2, Omicron, and Delta variants

**Fig 1:**
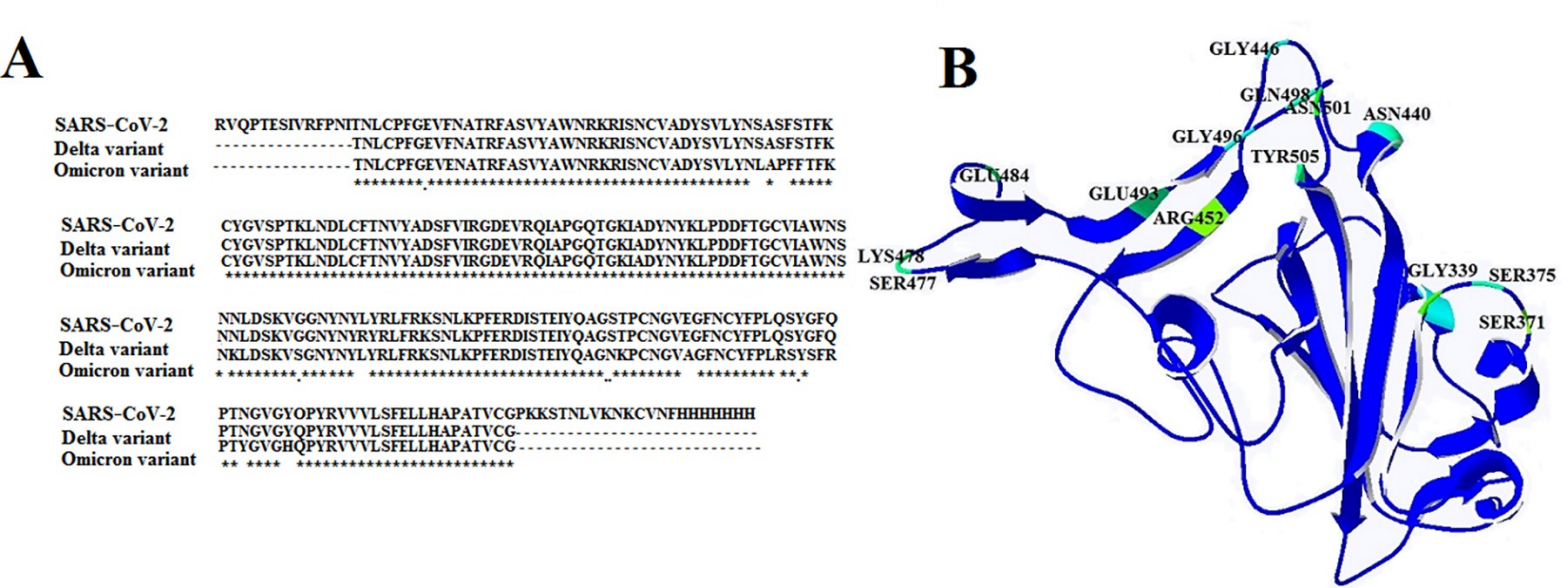
Sequence alignment of SARS-CoV-2, Omicron, and Delta variants (A). Location of mutations in RBD of virus structure (B).

Initially, differences in the genome sequences between SARS-CoV-2, Omicron, and Delta variants were compared and analyzed (Fig 1 and S1 Table). The results indicated that most mutations occurred in the receptor-binding motif (RBM) of SARS-CoV-2 and play an essential role in spike-receptor interactions.

### Analysis of binding free energy

**Fig 2:**
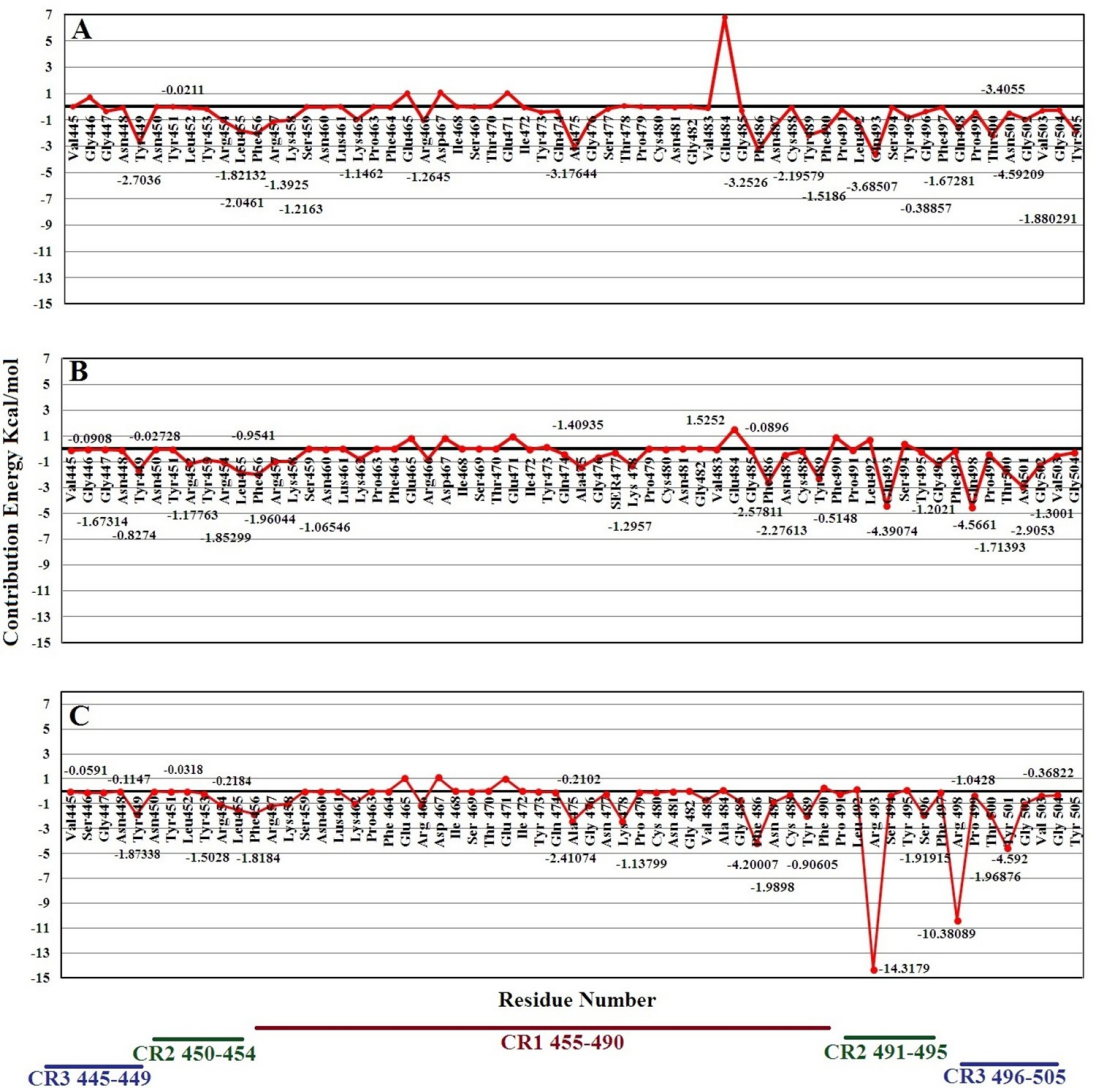
The free energy decomposition of the spike protein residues in the spike-ACE2 complex: (A) SARS-COV2, (B) Delta, and (C) Omicron variants.

Binding free energy analysis was performed for three complexes of SARS-CoV-2, namely SARS-CoV-2, Omicron, and Delta variants, and the obtained results are depicted in Table 1. The Molecular Mechanics Poisson–Boltzmann Surface Area (MM-PBSA) method was utilized to calculate the binding energies of the three variants of SARS-CoV-2. The Omicron variant had the lowest binding energy of -95.6496± 0.7364 kcal.mol^−1^. Notably, the electrostatic interactions play an essential role in binding affinities between SARS-CoV-2 and its cognate receptor. The binding free energy for the Delta variant was -81.2079± 0.7013 kcal.mol^−1^, which was lower compared to the wild type SARS-CoV-2. The highest binding free energy was detected with the SARS-CoV-2 variant with -69.2554± 0.6696 kcal.mol^−1^. It seems that some mutations in the RBM might play a crucial role in increasing the affinity of Delta and Omicron variants to ACE2. More details on the interaction between the spike protein and ACE2 is seen with the free energy decomposition analysis performed for the three complexes (Fig 2).

**Table 1:** The binding energies (kcal.mol^−1^) of the three variants of SARS-CoV-2 when bound to ACE2.

Variants of SARS-CoV-2	ΔG-bind	ENPOLAR	EGB	EEL	VDWAALS
**SARS-CoV-2**	**-69.2554± 0.6696**	**-10.9821**	**694.8892**	**-656.3850**	**-96.7775**
**Delta**	**-81.2079± 0.7013**	**-11.5792**	**1222.641**	**-1197.0871**	**-95.1829**
**Omicron**	**-95.6496± 0.7364**	**-11.4915**	**1754.392**	**-1740.7825**	**-97.7676**

### Free energy decomposition analysis

This analysis helps find the contribution of a single residue by summing its interactions across all residues. We divided the RBM of the spike into three sections, CR1 (residues 455–490), CR2 (residues 450–454 and 491–495), and CR3 (residues 445–449 and 496–506), to understand more about the specific interactions between the spike protein and ACE2 in each of these complexes (S2 Fig). These three regions are involved in the interaction of SARS-CoV-2 with the ACE2 receptor. Mutations were found in all three regions; however, most differences in free energy decomposition were observed in two regions containing residues 491–495 and 496–505 (CR2 and CR3, respectively). The free energy decomposition of Gln484 in the CR1 region also showed a significant difference between the three variants. The amount of unfavorable free energy for Gln484 in SARS-CoV-2 was 6.79417±0.142 kcal.mol^−1^, which was decreased to 1.5252±0.0629 kcal.mol^−1^ in the Delta variant. In the Omicron variant, this residue was mutated to Ala with a free energy decomposition of 0.09169± 0.0214 kcal.mol^−1^. These changes in the decomposition of Gln484 in total binding free energy might explain its critical role in the interaction of CR1 with ACE2. Steered molecular dynamic (SMD) simulation was applied to understand more detail on the role of mutations in the interaction between the spike protein and the ACE2 receptor.

### SMD simulation analysis

**Fig 3:**
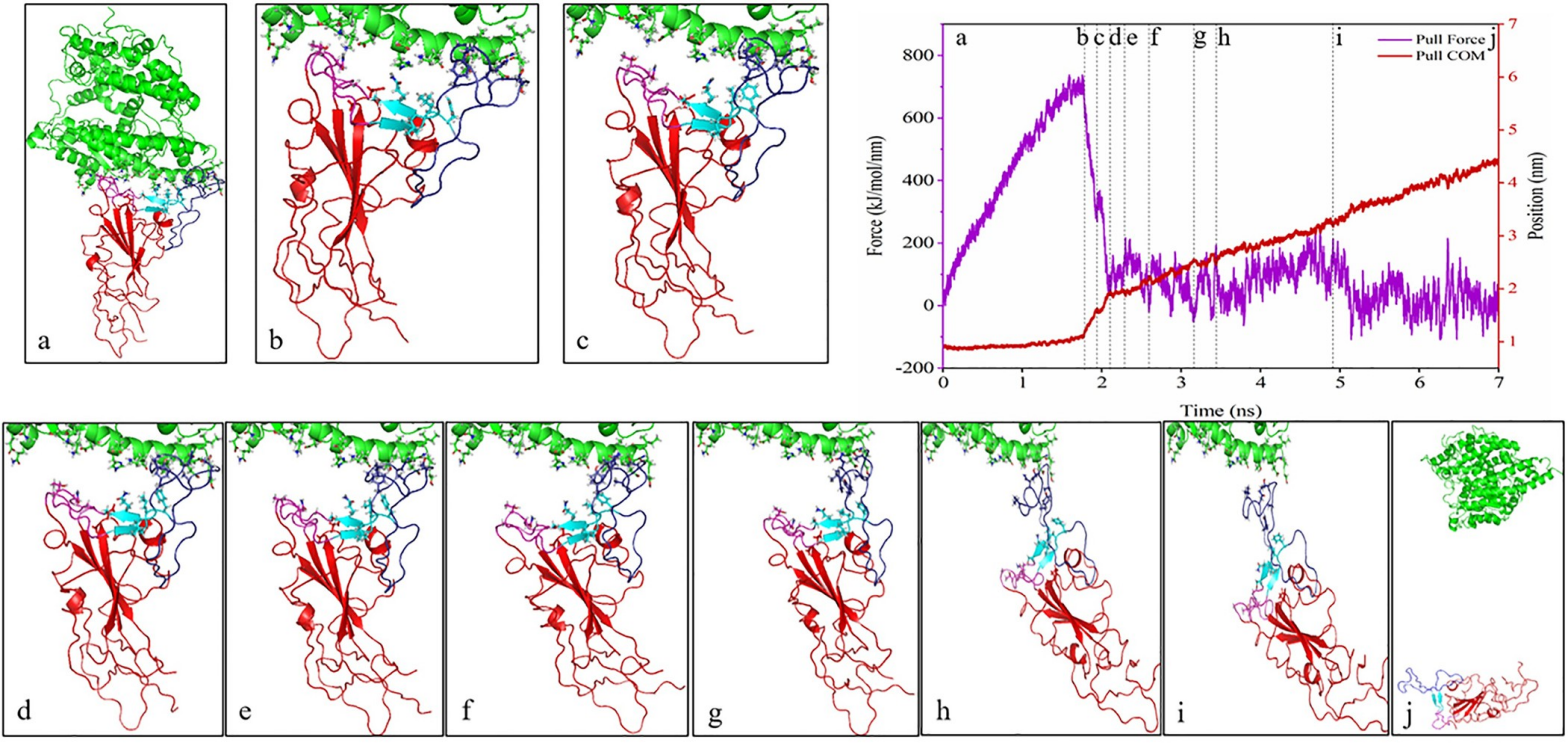
The pulling forces of the RBD of SARS-CoV-2 during 7 ns SMD simulations time along the Y-direction (purple graph), the distance between the centers of masses (COM) of selected ACE2 and RBD residues (red graph), and the marked lines represent the conformational state of RBD and ACE2 during SMD simulations. The dissociation process has been depicted from snapshots “a” to “j”.

**Fig 4:**
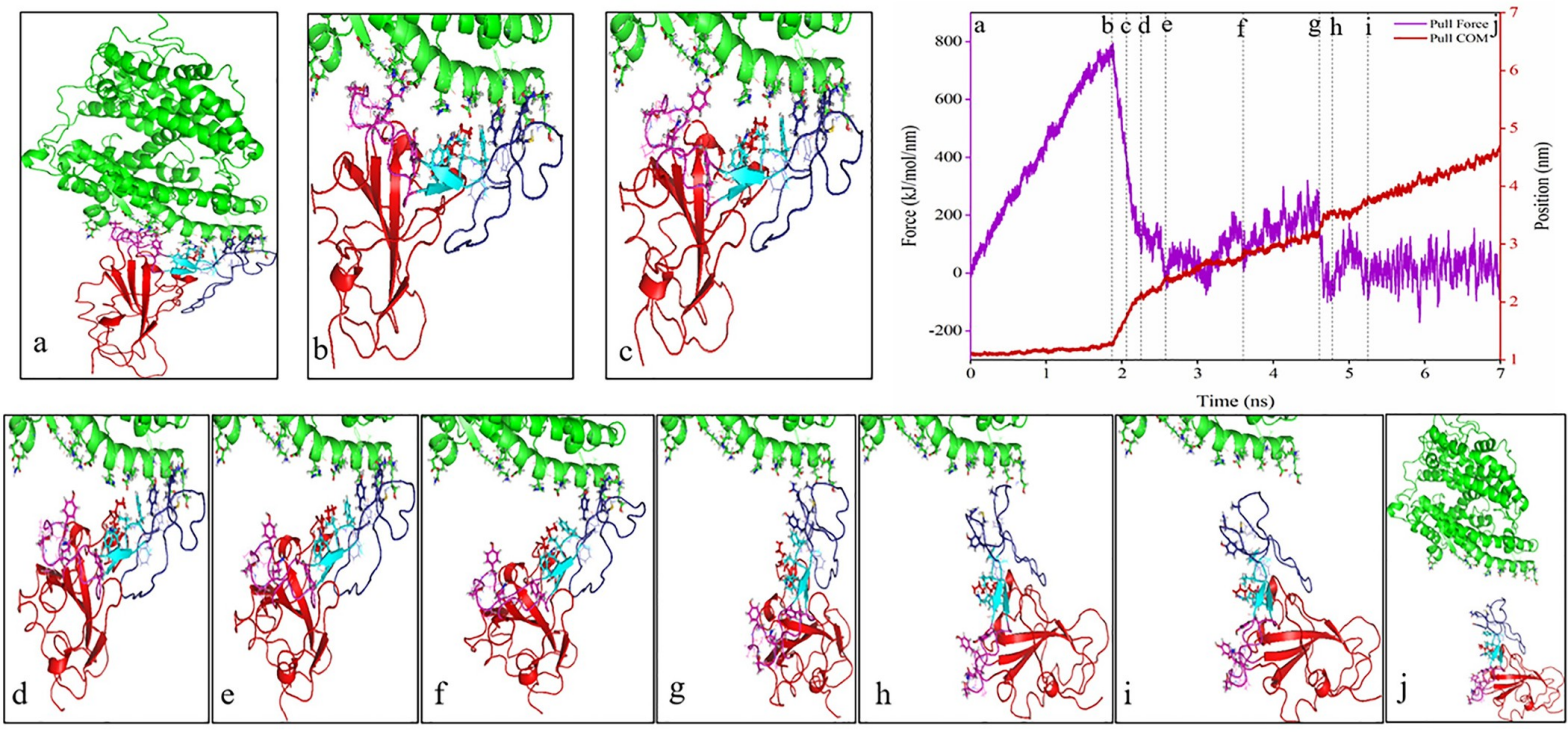
The pulling forces of the RBD of the Delta variant during 7 ns SMD simulations time along the Y-direction (purple graph), the distance between the centers of masses (COM) of selected ACE2 and RBD residues (red graph), and the marked lines represent the conformational state of RBD and ACE2 during SMD simulations. The dissociation process has been displayed from snapshots “a” to “j”.

**Fig 5:**
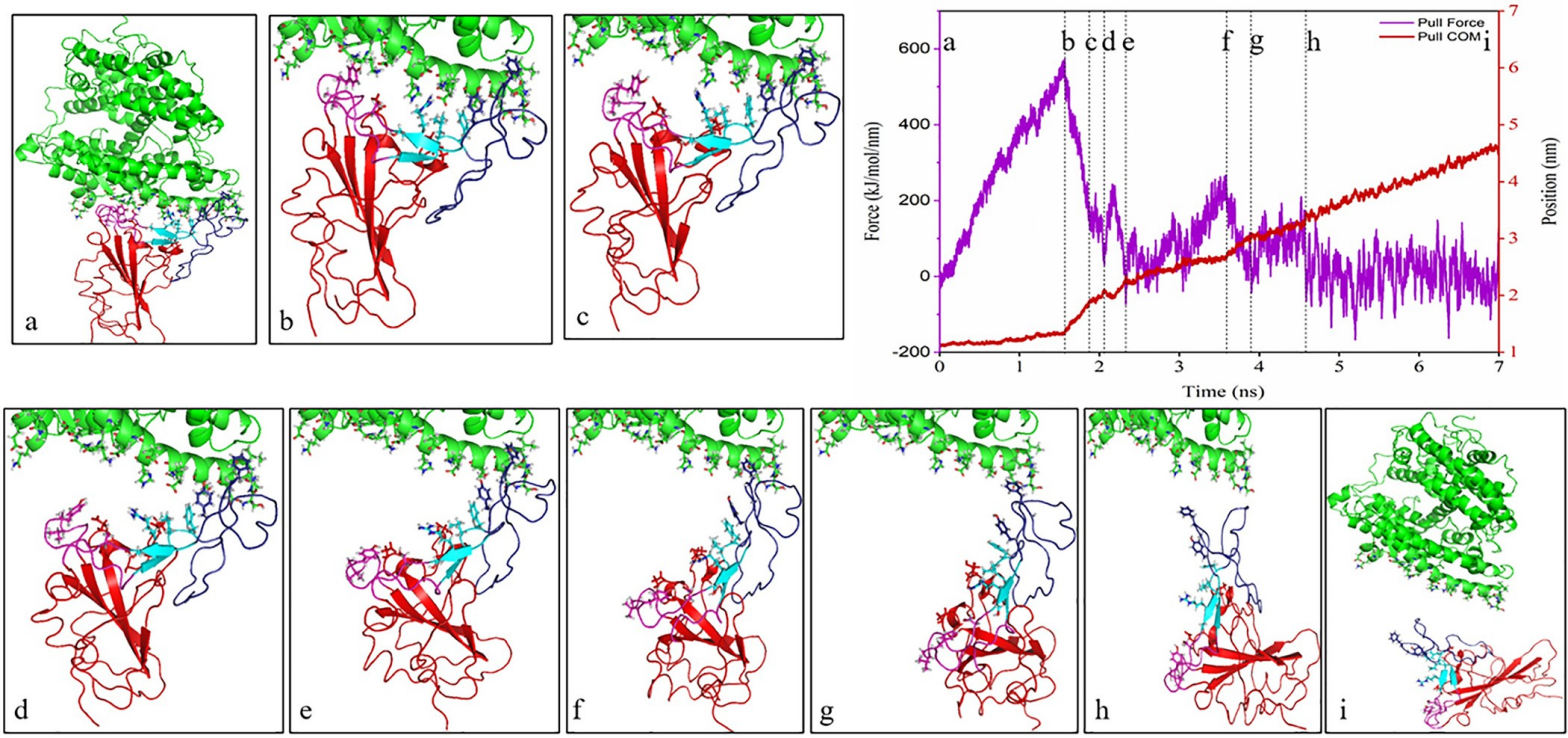
The pulling forces the RBD of the Delta variant during 7 ns SMD simulations time along the Y-direction (purple graph), the distance between the centers of masses (COM) of selected ACE2 and RBD residues (red graph), and the marked lines represent the conformational state of the RBD and ACE2 receptor during SMD simulations. The dissociation process has been shown from snapshots “a” to “i”.

In the SMD simulation, the spike glycoprotein was pulled out against the ACE2 receptor. The residues involved in the interaction between ACE2 and RBD are exhibited in S2 Table. In the pulling step, RBD residues, including K417-I418, G446-F456, Y473-A475, and N487-Y505, were pulled out against ACE2 residues, including S19-S43, T78-P84, Q325-N330, G352-I358, and P389-R393. The results of the SMD simulation showed that the amount of initial force necessary for the initiation of the dissociation process was higher for SARS-CoV-2 and Delta variants (740 kJ/mol/nm and 780 kJ/mol/nm, respectively) compared to the Omicron variant (590 kJ/mol/nm). CR2 (residues 450–454 and 491–495) and CR3 (residues 445–449 and 496–506) regions from SARS-CoV-2 and Delta variants were initially dissociated from the ACE2 receptor (Figs 3A–3D, 4A–4D and 5A–5D). The results also revealed that the CR1 region (residues 455–490) was the last region that retained the interaction between RBD and ACE2 during the SMD simulation (Figs 3E–3I, 4E–4H and 5E–5H). A minor peak is also visible for the initiation of CR1 dissociation, which is above 200 kJ/mol/nm for both Delta and Omicron variants, while such a force value for SARS-CoV-2 is gradually increased up to 200 kJ/mol/nm prior to dissociation. Of note, interactions in the CR2 and CR3 regions initially dissociated, and the spike-ACE2 complex remained bound as a result of CR1 interactions until the complex was completely dissociated.

**Fig 6:**
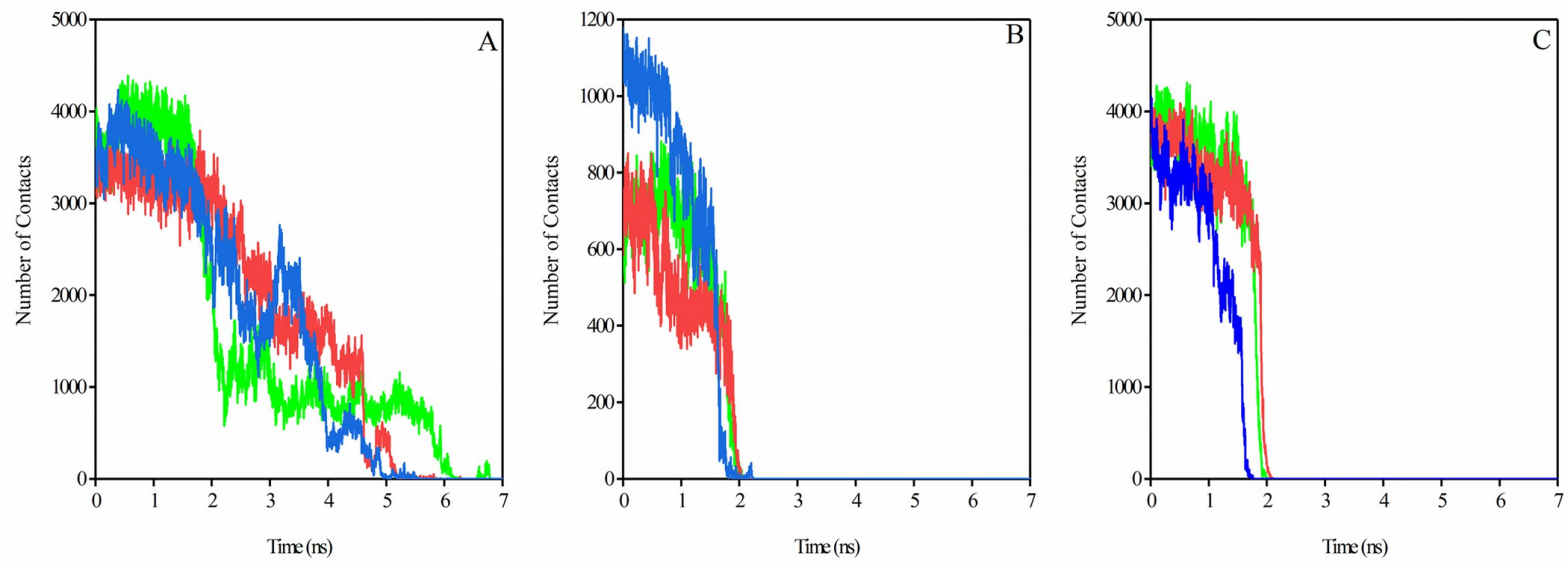
The number of total contacts between the residues of the ACE2 receptor with CR1 (A), CR2 (B), and CR3 (C) regions of the RBD during 7 ns of SMD simulations; SARS-CoV-2 (green color), Delta (red color), and Omicron (blue color) variants.

**Fig 7:**
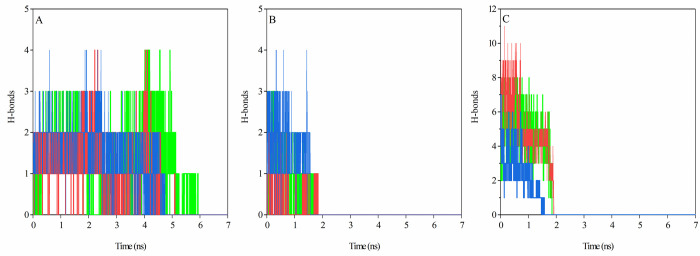
The number of H- bonds between ACE2 residues with CR1 (A), CR2 (B), and CR3 (C) of the RBD during 7 ns of SMD simulations; SARS-CoV-2 (green color), Delta (red color), and Omicron (blue color) variants.

The number of contacts, as well as secondary structure elements, was analyzed in these regions. The number of total contacts and number of H-bonds between spike-ACE2 complex inCR1, CR2, and CR3 regions during SMD simulation also verifies the dissociation process, as shown in the pulling force graph. Based on the total contacts and H-bond graphs (Figs 6 and 7), CR2 and CR3 regions were dissociated around 2 ns of the simulation, whereas CR1 remained connected for longer during the simulation. The CR2 region of the Omicron variant had higher initial total contacts and H-bonds compared to the SARS-CoV-2 and Delta variants (Figs 6B and 7B). In contrast, the Omicron variant had fewer initial total contacts and H-bonds in the CR3 region (Figs 6C and 7C), and these contacts were decreased earlier than SARS-CoV-2 and Delta variants. The number of contacts and H-bonds during SMD simulation in the CR1 region in the three complexes verified that this region remained bound to the ACE2 receptor for up to 5 ns in Delta and Omicron variants and 6 ns in the SARS-CoV-2 variant. Our results showed that CR1 had a high number of contacts and H-bonds with the ACE2 receptor in the three structures and thus may play critical roles in the interactions of the virus with its cognate receptor.

**Fig 8:**
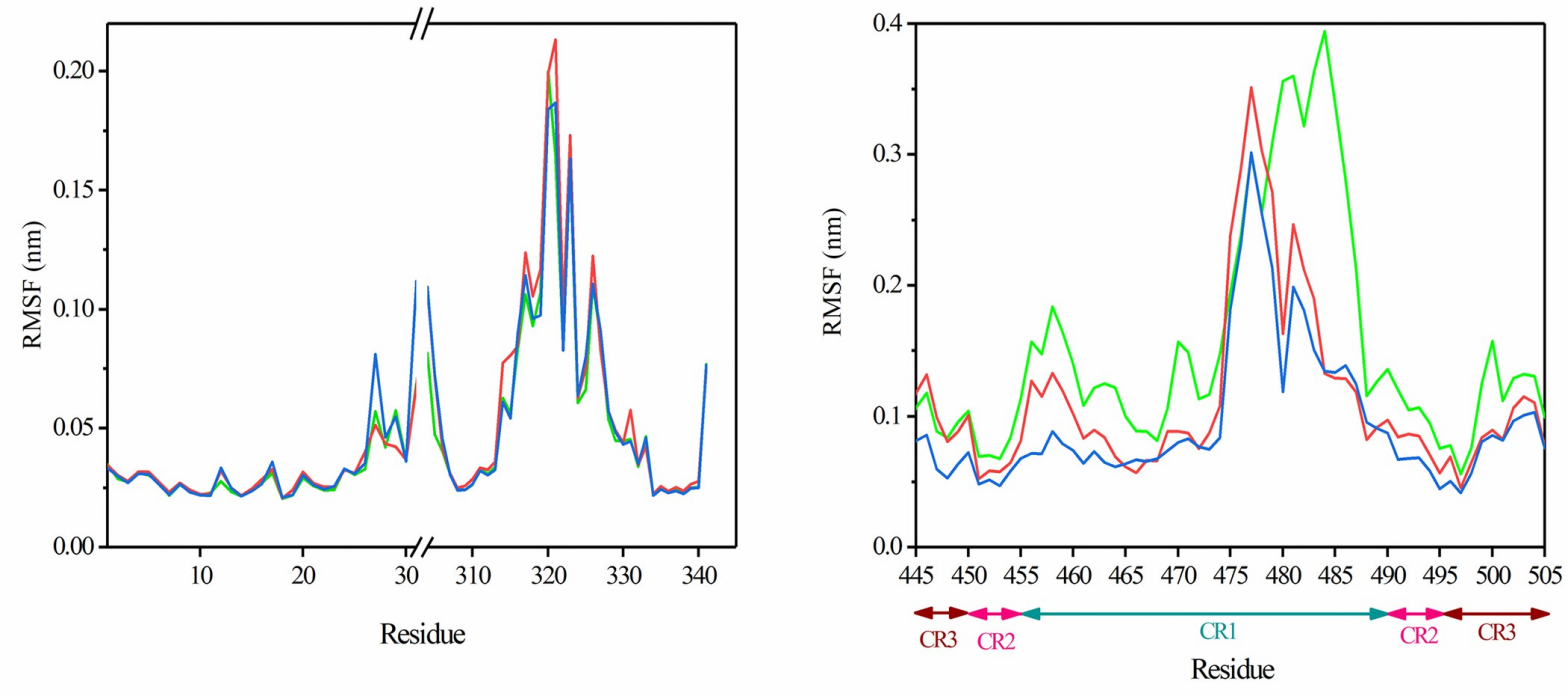
The RMSF values of Cα atoms of residues involved in the interaction of ACE2 with the virus during SMD simulation (A) and RBD of the spike protein (B); SARS-CoV-2 (green), Delta (red), and Omicron (blue) variants.

RMSF values of spike and ACE2 were obtained and are represented in Fig 8. According to RMSF graphs, local flexibilities for ACE2 were almost the same except for regions 27–30 for Omicron and 320 for Delta variants. The CR1 region of the three structures showed more flexibility than that of the CR2 and CR3 regions. The RBD of SARS-CoV-2 was more flexible than Delta and Omicron variants. The flexibility of CR1 may explain the resistance of this area during the dissociation process, as this region maintains its interaction with ACE2 for a longer simulation time. Also, the higher flexibility of CR1 in SARS-CoV-2 could explain how this region retains its contact until 6 ns of simulation, while CR1 of Delta and Omicron variants maintain their contact until 5 ns of simulation.

**Fig 9:**
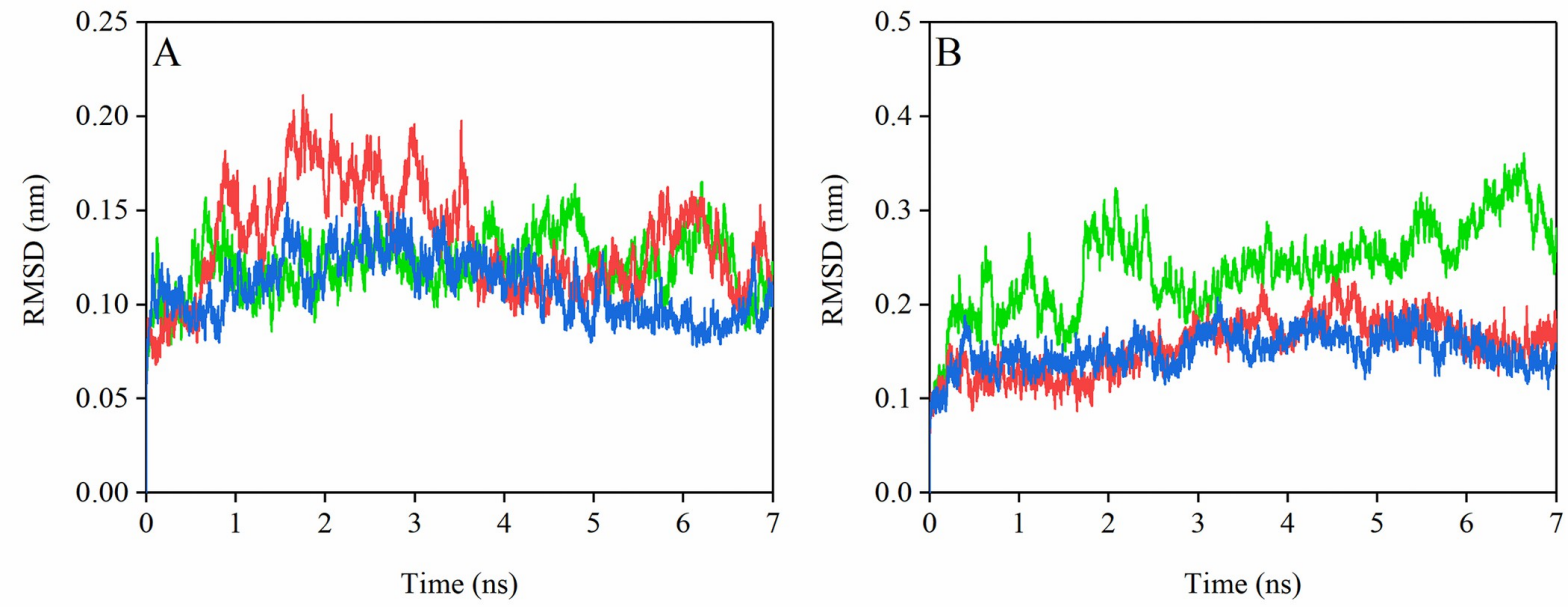
The RMSD of the backbone carbon atoms of selected ACE2 residues during SMD simulations (A) and RBD of the spike protein (B); SARS-CoV-2 (green), Delta (red), and Omicron (blue) variants.

Additionally, the secondary structure contents of the RBD have been analyzed within 7 ns of SMD simulation (S1 Fig). The results showed that the secondary structure in the CR1 region (S1A, S1D, and S1G Fig) was different between the three variants. The biggest difference in the CR1 region was observed in residues 473–475, 480–485, and 485–490. In residues 473–475, the bridge structure in the Delta variant was more than the two other variants. The secondary structure in residues 480–485 of the Delta and Omicron variants were similar to each other, while it was different for the wild type. In residues 485–490 of the wild type, the most frequent structure was detected to be the “turn” structure, while it was the “bridge” structure in the Delta variant. In this region, the frequency of the B-bridge structure in the Delta and Omicron variants was higher compared to the SARS-CoV-2 wild type. The secondary structure in the CR2 region (S1B, S1E, and S1H Fig) of the three variants was similar. In the CR3 region (S1C, S1F, and S1I Fig), the secondary structure of wild type and Omicron variants were similar, while it was different for the Delta variant. Our results indicated that the affinity of the Omicron variant for the ACE2 receptor was greater than Delta and SARS-CoV-2 variants, but the stability of the spike-ACE2 complex against dissociation was lower than that of the others. Moreover, we showed that the CR1 region plays a critical role in the interaction between the spike protein and ACE2. The RMSD graphs also verify that the Omicron variant had the lowest conformational motions during the SMD simulation (Fig 9).

**Fig 10:**
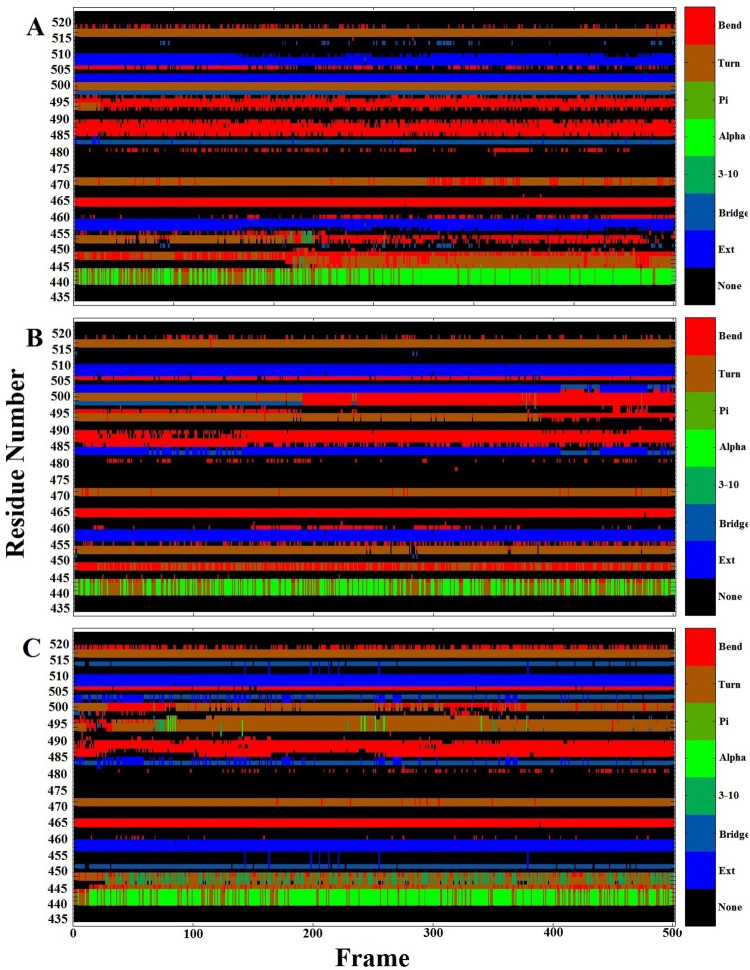
The analysis of the secondary structure contents of spikes during 100 ns of MD simulation. The secondary structures analysis of the SARS-CoV-2 (A). The secondary structure analysis of the Delta variant (B). The secondary structure analysis of the Omicron variant (C).

Finally, secondary structure analysis was carried out using the DSSP method in order to track all changes during 100 ns of MD simulation (Fig 10) for the three structures. Our results showed that the secondary structure contents of RBD had local differences between the three structures. For example, the secondary structure content in the CR3 region (residue 440–450) of the Omicron variant was mostly α-helix, but for Delta and SARS-CoV-2 variants was “bend.” Also, in the Omicron variants, there was a “bridge” structure in CR2 (residues 450–455). The secondary structure content of CR1 (residue 482–486) was "bridge" in the SARS-CoV2 and Omicron variants, but it was an “ext” structure in the Delta variant. The secondary structure in CR3 (residue 495–510) was different between the three variants. In this region, there is a "bridge" structure that is converted into a “bend” structure in the Delta variant during the simulation process.

### Protein structure and flexibility

**Fig 11:**
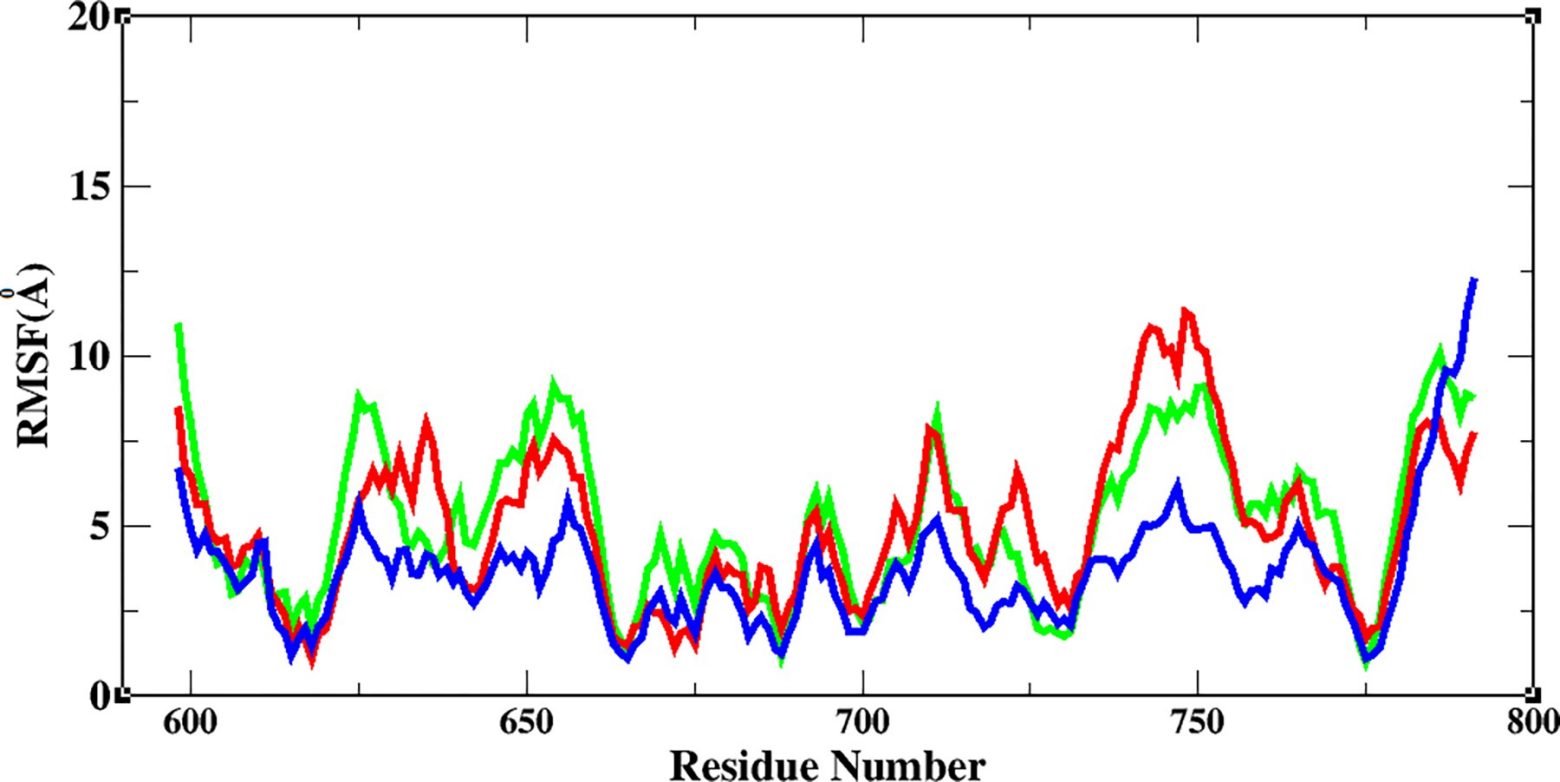
The RMSF values of Cα atoms in three variants. Wild type (green line), Delta (red line), and Omicron (blue line) variants.

In the last step, the flexibility of the spike protein was analyzed during 100 ns MD simulation. According to the RMSF graph (Fig 11), flexible regions and their patterns differed between the three variants. The results showed that the overall flexibility of the spike protein was higher in Delta and SARS-CoV-2 variants when compared to the Omicron variant. The highest flexibility in the RBM of the three variants was observed in CR1 (residues 443–487), which was related to the Delta variant. Based on the SMD results, the CR1 region was the last part to be dissociated from the ACE2 receptor, and the dissociation energy for this part was lower for the Omicron variant compared to other variants. According to our results, the amount of flexibility in the three variants play roles in the interaction between spike-ACE2. Also, the RMSD graph for the three variants during simulation (S3 Fig) shows that the Omicron variant had a higher RMSD value than the wild type and Delta variants.

To study the effect of mutations on the overall spike structure and flexibility, free and landscape (FEL), principal component (PCA) and dynamic cross-correlation matrix (DCCM) analyses have been performed (S4 Fig). Based on the FEL and PCA results (S4A1, S4B1 and S4C1 and S4A2, S4B2 and S4C2 Fig), the functional motions and energy landscape of the Omicron and Delta variants were different from SARS-CoV-2. The differences in the conformational and local motions of the Delta variant were more considerable that the Omicron. The results from DCCM also showed that these mutations affected on the global motions of the spike, as the correlation patterns in SARS-CoV-2 (residues 420–430 and 455–465 in CR1 region) have changed in the Delta and Omicron variants.

## Discussion

According to epidemiological studies, more than 5 million deaths are thought to be related to severe acute respiratory syndrome coronavirus 2 (SARS-CoV-2). SARS-CoV-2 efficiently uses several human host factors for viral attachment and entry [13]. The spike protein of SARS-CoV-2 can attach to the host cell receptor, human angiotensin-converting enzyme 2 (ACE2), more efficiently than the other closely related coronaviruses, such as SARS and MERS [14]. This newly discovered coronavirus has an unusually large number of mutations, several of which are novel and a significant number of which affect the spike glycoprotein [15].

Some of the mutations occurring in this variant are similar to other variants, while a number of mutations are also unique to this variant, responsible for the rapid transmission and virulence of the virus [16].

Another variant of SARS-CoV-2 that causes COVID-19 is known as the Omicron type. It was first reported to the World Health Organization (WHO) on 24 November from South Africa [17]. On 26 November 2021, the WHO designated it as VOC and then named it "Omicron,” the fifteenth letter of the Greek alphabet [18]. The Omicron variant is much more transmissible than previous variants of SARS-CoV-2, such as the Delta variant [19]. Therefore, analyzing the interaction of the ACE2 receptor with the Delta and Omicron variants of SARS-CoV-2 is necessary to discover the role of mutations in increasing the affinity of the virus to its host receptor.

In this study, we employed *in silico* methods to compare the interaction of the spike protein of SARS-CoV-2, Delta, and Omicron variants with the ACE2 receptor in order to discover the role of mutations in the biological process by which the virus infects the host cells. The MM-PBSA method was applied to compare the free energy between the three complexes. Our results indicated that the highest and lowest binding affinities of spike for ACE2 were related to Omicron and SARS-CoV-2 variants, respectively. Electrostatic interactions play the most significant role in the interaction between the virus and the ACE2 receptor in three forms of the complex. Pitsillou et al. reported that the Delta and Omicron variants bind to the ACE2 receptor with similar affinities (-39.4 and -43.3 kcal.mol^-1^, respectively), and their binding affinity was stronger than the SARS-CoV-2 variant (-33.5 kcal.mol^-1^) [20]. Khan and colleagues indicated that the Omicron variant had the highest binding affinity (-52.50 kcal.mol^-1^) for the ACE2 receptor. However, consistent with our findings, these values were -29.21 kcal.mol^-1^ and -26.62 kcal.mol^-1^ for the Delta variant and SARS-CoV-2 variants, respectively [21].

Henrique et al. demonstrated that mutations in the RBD of the Omicron variant accelerated the affinity of spike protein for ACE2 receptor and may explain the high transmissibility of this variant in comparison with other SARS-CoV-2 variants. In agreement with our results, the stabilization effect of the RBD–ACE2 receptor complex of the Omicron variant is mainly due to the substitution of uncharged residues by positively charged residues (Lys and Arg) in a key position [22].

The values of free energy decomposition for all residues in binding regions of the spike protein and receptor were analyzed. The RBD has two subdomains, including a core structure formed by a five-stranded antiparallel β-sheets covered with short connecting α-helices on both sides, and an extended loop named the ‘receptor-binding motif’ (RBM), which wraps around one edge of the core structure and makes all the contacts with ACE2. In our previous study, we reported that there are three regions in the receptor-binding motif that are involved in the spike-ACE2 interaction. Two regions were located at the beginning and the end of the receptor-binding motif. The third region was located in the middle area of the receptor-binding motif of the spike protein, and most mutations occur in these regions [9]. Based on the interaction of the receptor-binding motif with the ACE2 receptor, this region is divided into three parts, namely CR1 (residues 455–490), CR2 (residues 450–454 and 491–495), and CR3 (residues 445–449 and 496–506). Several differences in the interaction pattern between the spike protein and ACE2 receptor have been observed in the three complexes. The most significant difference was detected in two regions, including residues 491–495 and 496–505, located in the CR2 and CR3 regions, respectively, and Gln484 in CR1. In fact, several mutations in the receptor-binding motif of the spike protein led to a change in the binding free energy contribution of each residue in the interaction between the ACE2 receptor and the spike protein of SARS-CoV-2, Delta, and Omicron variants. Da Costa et al. indicated that a number of mutations, including N440K, T478K, Q493R, and Q498R in the RBD of the Omicron variant, cause favorable interactions between the RBD and ACE2 receptor [23].

In the next step, the dissociation process of the spike-ACE2 complex for three complexes was investigated using SMD simulation in order to assess the role of each residue in maintaining the interaction until each complex was completely detached. According to our results, the amount of pulling force to initiate the dissociation process in SARS-CoV-2 and Delta variants during SMD simulation was nearly the same, and this force was higher than that of the Omicron variant. Abidi et al. reported that the dissociation force of RBD-ACE2 for SARS-CoV-2 and Delta variants were close to each other [24].

Nguyen et al. also reported that the Omicron variant binds to hACE2 more strongly than the SARS-CoV-2 variant [25]. The results of SMD showed that the SARS-CoV2 and Delta variants exhibit more resistance to dissociation from the ACE2 receptor. This result also revealed that the interactions in the RBD-ACE2 receptor complex remained stable by the CR1 region during SMD simulation. In fact, other interactions (CR2, CR3) were broken at first, and the interactions in this region continuously remained until the complexes were totally dissociated. It seems that CR1 plays a critical role in the interactions between the ACE2 receptor and spike protein, but most mutations were observed in the CR2 and CR3 regions (residues 491–495 and 496–505). These mutations led to a decrease in the binding free energy of residues 491–495 and 496–505 in Omicron and Delta variants when compared to SARS-CoV-2. However, based on the SMD results, the amount of pulling force in the SARS-CoV-2 and Delta variants were higher than that of the Omicron variant. We investigated the flexibility of the spike structure in the three complexes to determine the cause of these events. According to these results, the flexibility of these regions was different in SARS-CoV-2, Delta and Omicron variants. In general, proteins have dynamic structures, and their biological functions are dependent on this flexibility and allowing proteins to change their conformation to respond to the presence of other molecules and/or variations in the environment [26]. Biochemical processes, such as signal transduction, antigen recognition, protein transport, and enzyme catalysis, rely on this ability to change conformation. The adaptability and flexibility of a protein may result in either subtle change, such as when a few amino acid side chains of an enzyme move to bind a small substrate, or in more dramatic changes, such as when a large conformation of a protein is triggered by the presence of a ligand [26].

According to the above statements, and consistent with our findings, the flexibility of the spike protein plays an important role in the interaction between the spike protein and the ACE2 receptor. Based on our results, the Omicron variant possesses the highest binding affinity for the ACE2 receptor compared to other variants that have been confirmed in other studies but were easily dissociated during SMD. In this study, we demonstrated that the flexibility of the spike protein in the Omicron variant was less than other variants, explaining the behavior of this structure in protein-receptor interaction. The SMD analysis (RMSF and RMSD) also revealed that the spike structure in the Omicron variant had the lowest flexibility compared to the other variants. According to the SMD results, the most flexible part in tree variants also was in the CR1 region, which is similar to the MD results. It seems that the flexibility of this region effectively contributes to the interaction between the spike protein and the ACE2 receptor. Also, the more flexible CR1 tends to remain in the receptor interaction longer during the SMD simulation. Also, FEL and PCA results showed that the Delta variant had more flexible structure during the simulation.

In addition, the lowest flexibility and binding free energy was related to the Omicron variant, while the strongest interaction pertained to the SARS-CoV-2 and Delta variants. The binding affinity in SARS-CoV-2 and Delta variants was lower and could be related to their conformational entropy. In other words, higher flexibility in SARS-CoV-2 and Delta structure leads to an increase in structural entropy which contribute to a more positive binding free energy [27]. Therefore, the lowest free binding energy and highest affinity were related to the Omicron variant.

## Conclusion

In this study, we evaluated the effect of mutations on structural and binding free energies in the RBD of the spike protein in three variants of SARS-CoV-2, namely Delta, Omicron and SARS-COV-2 wild type in complex with the human ACE2 receptor. The binding affinity of each RBD for the ACE2 receptor was obtained using the MM-PBSA method. The results showed that the trends in the calculated binding free energies were well correlated with affinity of each viral variant to ACE2 receptor. The mutation in the RBD of the Omicron variant increases the affinity of the spike protein for the ACE2 receptor and may explain its high transmissibility in comparison with other SARS-CoV-2 variants. In this study, we also indicated that the flexibility of the spike protein in the Omicron variant was less than in other variants, and suggest that this could be the reason for the spike protein behavior in the protein-receptor interaction.

## Methods

### Sequence and structural analysis of Spike and ACE2 receptor

Crystal structures of the SARS-CoV-2 spike receptor-binding domain bound to ACE2 receptor (PDB code: 6m0j) were obtained from Protein Data Bank (https://www.rcsb.org), and mutations were extracted from the SND server (http://www-cryst.bioc.cam.ac.uk/~sdm/sdm.php) to obtain Delta and Omicron variants. *In silico* studies were then carried out on three variants to examine the significance of mutations in the receptor-binding motif (RBM) or other regions of the receptor-binding domain (RBD), as well as the spike affinity for the ACE2 receptors to better understand the viral affinity to ACE2. The structural analysis was conducted by Swiss-PDB viewer 4.0.1 and Pymol 1.3 [28, 29]. The FASTA files of variants were obtained from Protein Data Bank and aligned by ClustalW (https://www.ebi.ac.uk/Tools/msa/clustalo).

### Molecular dynamics simulation

The molecular dynamics simulation of complex structures was performed to understand the interaction between the spike and its receptor using AMBER20 [30] and pmemd.cuda GPU code and ff14SB force-field [31]. The complex was neutralized by adding Cl^-^ ions to structures using the LEaP module. Afterward, the structures were immersed in a truncated octahedron box filled with a 10 Å layer of TIP3P water molecules [30]. Then, the topology and coordination were saved for the subsequent steps in simulations. The energy minimizing of the solvated spike-ACE2 complex was performed within two phases. First, the ions and water molecules were minimized by 3000 steps; then, the entire system was minimized by 5,000 steps employing the steepest-decent and conjugate gradient algorithms. In the periodic boundary condition, the cutoff distance was adjusted to 10 Å to calculate non-covalent interactions by the PME. The system was heated from 0 to 300 K for 200 ps, with the NVT ensemble using a Langevin thermostat at a collision frequency of 2 ps [32]. The bonds, including hydrogen atoms, were constrained using the SHAKE algorithm [33]. Before implementation of the MD simulation, the equilibration in the NPT group was performed for 1 ns with a Berendsen barostat and a relaxation time of 2 ps, and the pressure was set to 1 atm. Ultimately, the MD simulation was performed for 100 ns with the NPT ensemble. The time-step was set at 2 fs, and the coordinates were stored every 0.8 ps.

### Trajectory analysis

The trajectory analysis was carried out using CPPTRAJ [34] and PYTRAJ [34] from AmberTools 20 to analyze the trajectories.

### Molecular mechanics Poisson–Boltzmann surface area (MM-PBSA) calculation

In order to compare the binding affinity of the spike-ACE2 complex in three variants (PDB code 6m0j), MM-PBSA calculation was conducted with previous procedures by Amber 20 for 100 ns using ff99SB force-field. The analysis of binding free energies was performed by mmpbsa.py [35].

### Steered molecular dynamic (SMD) simulation

SMD simulations were applied to explore key RBD interaction residues with the ACE2 receptor. The initial structures for pulling simulations were obtained from Classical Molecular Dynamics (CMD) simulations. The simulation box of each complex was changed to a dimension of 15 × 35 × 15 nm. Water molecules were added, and minimization and equilibration steps were performed for each system again. At first, 50,000 steps of energy minimization were carried out using the steepest descent algorithm. All complexes were equilibrated in NVT, and then the NPT ensemble was used to equilibrate each complex for 1 ns individually while placing a restraint on the protein backbone atoms. The SMD simulations were carried out with the harmonic sparing constant of 1000 kJ.mol^-1^.nm^-2^ and the pulling rate of 0.0005 nmps^-1^. In the pulling out step, the atoms of several RBD residues, including K417-I418, G446-F456, Y473-A475, and N487-Y505, were pulled out against atoms of ACE2 residues S19-S43, T78-P84, Q325-N330, G352-I358, and P389-R393. The mentioned ACE2 receptor residues were kept fixed during the SMD simulations. The pulling direction of RBD was conducted along the Y-direction. All SMD simulations were performed for 7ns using Gromacs 2022.2 employing CHARMM force-field parameters 20 [36, 37].

## Supporting information

S1 Table List of mutations in receptor-binding motif of SARS-CoV-2 compared to Delta variant and Omicron variant. (DOCX)

S2 Table The residues involved in the interaction between ACE2 and RBD. (DOCX)

S1 Fig Changes of the secondary structure elements of CR1 domain (A, D, and G), CR2 domain (B, E, and H), and CR3 domain (C, F, and I) during SMD simulations. SARS-CoV-2 (A, B and C), Delta (D, E and F), and Omicron (G, H, and I).
(TIF)

S2 Fig Three regions in RBD domain of spike glycoproteins: Three regions were showed with red, yellow and blue that are CR1 (455–490), CR2 ((450–454 491–495)), CR3 ((445–449) (496–506)) respectively. (TIF)

S3 Fig RMSD the backbone carbon atoms of selected ACE2 residues and RBD domains. Wild Type- SARS-CoV2 (green color), Delta (red color), and Omicron (blue color).
(TIF)

S4 Fig Free energy landscape (FEL), principal component (PCA) and dynamic cross-correlation matrix (DCCM) analysis of spike protein. SARS-CoV-2 (A1, A2, A3), Delta (B1, B2, B3), and Omicron (C1, C2, C3) variants.
(TIF)

## References

[1] To KK, Tsang OT, Leung WS, et al. Temporal profiles of viral load in posterior oropharyngeal saliva samples and serum antibody responses during infection by SARS-CoV-2: an observational cohort study. Lancet Infect Dis. 2020; 20: 565–574. doi: 10.1016/S1473-3099(20)30196-1 32213337 PMC7158907

[2] Chan JF, Yuan S, Kok KH, et al. A familial cluster of pneumonia associated with the 2019 novel coronavirus indicating person-to-person transmission: a study of a family cluster. Lancet. 2020; 395: 514–523. doi: 10.1016/S0140-6736(20)30154-9 31986261 PMC7159286

[3] Letko M, Marzi A, Munster V. Functional assessment of cell entry and receptor usage for SARS-CoV-2 and other lineage B betacoronaviruses. Nat Microbiol. 2020; 5: 562–569. doi: 10.1038/s41564-020-0688-y 32094589 PMC7095430

[4] Dudas G, Hong SL, Potter BI, et al. Emergence and spread of SARS-CoV-2 lineage B.1.620 with variant of concern-like mutations and deletions. Nat Commun. 2021; 12: 5769. doi: 10.1038/s41467-021-26055-8 34599175 PMC8486757

[5] Pandey SC, Pande V, Sati D, Upreti S, Samant M. Vaccination strategies to combat novel corona virus SARS-CoV-2. Life Sci. 2020; 256: 117956. doi: 10.1016/j.lfs.2020.117956 32535078 PMC7289747

[6] Kumar S, Thambiraja TS, Karuppanan K, Subramaniam G. Omicron and Delta variant of SARS-CoV-2: A comparative computational study of spike protein. J Med Virol. 2022; 94: 1641–1649. doi: 10.1002/jmv.27526 34914115

[7] Sharma V, Rai H, Gautam DNS, Prajapati PK, Sharma R. Emerging evidence on Omicron (B.1.1.529) SARS-CoV-2 variant. J Med. Virol. 2022 May; 94(5): 1876–1885. doi: 10.1002/jmv.27626 35083761 PMC9015596

[8] Mahase E. Delta variant: what is happening with transmission, hospital admissions, and restrictions? BMJ. 2021; 373: n1513. doi: 10.1136/bmj.n1513 34130949

[9] Bian L, Gao Q, Gao F, et al. Impact of the Delta variant on vaccine efficacy and response strategies. Expert Rev Vaccines. 2021; 20: 1201–1209. doi: 10.1080/14760584.2021.1976153 34488546 PMC8442750

[10] Farzaneh Jafary, Jafari Sepideh, and Mohamad Reza Ganjalikhany. "In silico investigation of critical binding pattern in SARS-CoV-2 spike protein with angiotensin-converting enzyme 2." Scientific Reports. 2021; 11: 6927. doi: 10.1038/s41598-021-86380-2 33767306 PMC7994905

[11] Wu L, Zhou L, Mo M, Liu T, Wu C, Gong C, et al. SARS-CoV-2 Omicron RBD shows weaker binding affinity than the currently dominant Delta variant to human ACE2. Signal Transduct Target Ther. 2022; 7: 8. doi: 10.1038/s41392-021-00863-2 34987150 PMC8727475

[12] Kumar S, Thambiraja TS, Karuppanan K, Subramaniam G. Omicron and Delta variant of SARS-CoV-2: A comparative computational study of spike protein. J Med Virol. 2022; 94: 1641–1649. doi: 10.1002/jmv.27526 34914115

[13] To KK, Tsang OT, Leung WS, et al. Temporal profiles of viral load in posterior oropharyngeal saliva samples and serum antibody responses during infection by SARS-CoV-2: an observational cohort study. Lancet Infect Dis. 2020; 20: 565–574. doi: 10.1016/S1473-3099(20)30196-1 32213337 PMC7158907

[14] Letko M, Marzi A, Munster V. Functional assessment of cell entry and receptor usage for SARS-CoV-2 and other lineage B betacoronaviruses. Nat Microbiol. 2020; 5: 562–569. doi: 10.1038/s41564-020-0688-y 32094589 PMC7095430

[15] Harvey W.T., Carabelli A.M., Jackson B. et al. SARS-CoV-2 variants, spike mutations and immune escape. Nat Rev Microbiol. 2021; 19: 409–424. doi: 10.1038/s41579-021-00573-0 34075212 PMC8167834

[16] Riley S, Eales O, Haw D. REACT-1 round 13 interim report: acceleration of SARS-CoV-2 Delta epidemic in the community in England during late June and early July. medRxiv. 2021.

[17] Omicron (B.1.1.529) variant. Atlanta, GA: US Department of Health and Human Services, CDC; 2021.

[18] World Health Organization. Classification of Omicron (B.1.1.529): SARS-CoV-2 variant of concern. Geneva, Switzerland: World Health Organization. 2021; 3.

[19] Duong BV, Larpruenrudee P, Fang T, Hossain SI, Saha SC, Gu Y, et al. Is the SARS CoV-2 Omicron Variant Deadlier and More Transmissible Than Delta Variant? Int J Environ Res Public Health. 2022; 19: 4586. doi: 10.3390/ijerph19084586 35457468 PMC9032753

[20] Pitsillou E, Liang JJ, Beh RC, Hung A, Karagiannis TC. Molecular dynamics simulations highlight the altered binding landscape at the spike-ACE2 interface between the Delta and Omicron variants compared to the SARS-CoV-2 original strain. Comput Biol Med. 2022; 149: 106035. doi: 10.1016/j.compbiomed.2022.106035 36055162 PMC9420038

[21] Khan A, Khan SA, Zia K, Altowyan MS, Barakat A, Ul-Haq Z. Deciphering the Impact of Mutations on the Binding Efficacy of SARS-CoV-2 Omicron and Delta Variants With Human ACE2 Receptor. Front Chem. 2022; 10: 892093. doi: 10.3389/fchem.2022.892093 35755247 PMC9213841

[22] da Costa CHS, de Freitas CAB, Alves CN, Lameira J. Assessment of mutations on RBD in the Spike protein of SARS-CoV-2 Alpha, Delta and Omicron variants. Sci Rep. 2022; 12: 8540. doi: 10.1038/s41598-022-12479-9 35595778 PMC9121086

[23] Mohammadi M, Shayestehpour M, Mirzaei H. The impact of spike mutated variants of SARS-CoV2 [Alpha, Beta, Gamma, Delta, and Lambda] on the efficacy of subunit recombinant vaccines. Braz J Infect Dis. 2021 Jul-Aug;25(4):101606. doi: 10.1016/j.bjid.2021.101606 34428473 PMC8367756

[24] Abidi Mohadese, Soheilifard Reza, Ghasemi Reza Hasanzadeh. Comparison of the unbinding process of RBD-ACE2 complex between SARS-CoV-2 variants (Delta, Delta plus, and Lambda): A steered molecular dynamics simulation. Molecular Simulation. 2022; 48: 1–8.

[25] Nguyen HL, Thai NQ, Nguyen PH, Li MS. SARS-CoV-2 Omicron Variant Binds to Human Cells More Strongly than the Wild Type: Evidence from Molecular Dynamics Simulation. J Phys Chem B. 2022; 126: 4669–4678. doi: 10.1021/acs.jpcb.2c01048 35723978 PMC9235043

[26] Kaare Teilum Johan G. Olsen Birthe B. Kragelund, Functional aspects of protein flexibility. Cell. Mol. Life Sci. 2009; 66: 2231–2247. 19308324 10.1007/s00018-009-0014-6 PMC11115794

[27] Atkins Peter and de Paula Julio. Physical Chemistry for the Life Sciences. New York, NY: W. H. Freeman and Company. 2006; 286: 153–163,

[28] Guex N. & Peitsch M. C. SWISS-MODEL and the Swiss-PdbViewer: An environment for comparative protein modeling. Electrophoresis. 1997; 18: 2714–2723. doi: 10.1002/elps.1150181505 9504803

[29] PyMOL Molecular Graphics System. Delano Scientifc, San Carlos. (2002).

[30] Case D. A. et al. AMBER 2015, University of California, San Francisco (2015).

[31] James A. M, 28. et al. f14SB: Improving the accuracy of protein side chain and backbone parameters from f99SB. J. Chem. Teory Comput. 2015; 11: 3696–3713. 10.1021/acs.jctc.5b00255 PMC4821407 26574453

[32] Loncharich R. J., Brooks B. R. & Pastor R. W. Langevin dynamics of peptides: Te frictional dependence of isomerization rates of N-actylananyl-N’-methylamide. Biopolymers. 1992; 32: 523–535. 1515543 10.1002/bip.360320508

[33] Ryckaert J. P., Ciccotti G. & Berendsen H. J. C. Numerical integration of the Cartesian equations of motion of a system with constraints: Molecular dynamics of n-alkanes. J Comput Phys. 1977; 23: 327–341.

[34] Roe Daniel R. and Cheatham Thomas E. PTRAJ and CPPTRAJ: Software for Processing and Analysis of Molecular Dynamics Trajectory Data., Journal of Chemical Theory and Computation. 2013 9 (7), 3084–3095. doi: 10.1021/ct400341p 26583988

[35] Miller B. R. et al. MMPBSA.py: An Efficient Program for End-State Free Energy Calculations. J. Chem. Theory Comput. 2012; 8: 3314–3321. doi: 10.1021/ct300418h 26605738

[36] Mark James Abraham Teemu Murtola, Schulz Roland, Szilárd Páll Jeremy C. Smith, Hess Berk, et al., GROMACS: High performance molecular simulations through multi-level parallelism from laptops to supercomputers, SoftwareX. 2015; 1: 19–25.

[37] Vanommeslaeghe, Hatcher K., Acharya E, Kundu C, Zhong S, Shim S, Darian J. E., et al. "CHARMM General Force Field (CGenFF): A force field for drug-like molecules compatible with the CHARMM all-atom additive biological force fields," Journal of Computational Chemistry. 2010; 31: 671–90. 19575467 10.1002/jcc.21367 PMC2888302

