# Supplementary material for: Different aspects in explaining how mutations could affect the binding mechanism of receptor binding domain of SARS-CoV-2 spike protein in interaction with ACE2
Source: PLoS One. 2023 Sep 8;18(9):e0291210. doi: 10.1371/journal.pone.0291210 (PMC10490914; doi:10.1371/journal.pone.0291210)
Supplement: S1 Table — (DOCX) [file pone.0291210.s001.docx]

| SARS-CoV2 | Delta variant | Omicron variant |
| --- | --- | --- |
| Gly339 | **Gly339** | **Asp339** |
| Ser371 | **Ser371** | **Lys371** |
| Ser375 | **Ser375** | **Phe375** |
| Asn440 | **Asn440** | **Lys440** |
| Gly446 | **Gly446** | **Ser446** |
| Lue452 | **Arg452** | **Lue452** |
| Ser477 | **Ser477** | **Asn477** |
| Thr478 | **Lys478** | **Lys478** |
| Glu484 | **Glu484** | **Ala484** |
| Gln493 | **Gln493** | **Arg493** |
| Gly496 | **Gly496** | **Ser496** |
| Gln498 | **Gln498** | **Arg498** |
| Asn501 | **Asn501** | **Tyr501** |
| Tyr505 | **Tyr505** | **His505** |

**Table S1. List of mutations in receptor-binding motif of SARS-CoV-2 compared to Delta variant and Omicron variant.**
