# Supplementary material for: Different aspects in explaining how mutations could affect the binding mechanism of receptor binding domain of SARS-CoV-2 spike protein in interaction with ACE2
Source: PLoS One. 2023 Sep 8;18(9):e0291210. doi: 10.1371/journal.pone.0291210 (PMC10490914; doi:10.1371/journal.pone.0291210)
Supplement: S2 Table — (DOCX) [file pone.0291210.s002.docx]

**Table S2. The residues involved in the interaction between ACE2 and RBD.**

| SARS-CoV2 | | Delta | | Omicron | |
| --- | --- | --- | --- | --- | --- |
| ACE2 | **RBD** | **ACE2** | **RBD** | **ACE2** | **RBD** |
| SER 19 | **LYS 417** | **SER 19** | **GLY 446** | **SER 19** | **LYS 417** |
| GLN 24 | **GLY 446** | **GLU 23** | **TYR 449** | **GLN 24** | **SER 446** |
| THR 27 | **TYR 449** | **GLN 24** | **TYR 453** | **THR 27** | **TYR 449** |
| ASP 30 | **TYR 453** | **THR27** | **LYS 458** | **PHE 28** | **TYR 453** |
| LYS 31 | **TYR 473** | **ASP 30** | **TYR 473** | **ASP 30** | **TYR 473** |
| HIE 34 | **ALA 475** | **LYS 31** | **ALA 475** | **LYS 31** | **ALA 475** |
| GLU 35 | **SER 477** | **HIE 34** | **GLY 476** | **HIE 34** | **GLY 476** |
| GLU 37 | **GLU 484** | **GLU 35** | **SER 477** | **GLU 35** | **ASN 477** |
| ASP 38 | **GLY 485** | **GLU 37** | **ASN 487** | **GLU 37** | **ASN 487** |
| TYR 41 | **ASN 487** | **ASP 38** | **GLN 493** | **ASP 38** | **TYR 489** |
| GLN 42 | **TYR 489** | **TYR 41** | **GLY 496** | **TYR 41** | **ARG 493** |
| TYR 83 | **GLN 493** | **GLN 42** | **GLN 498** | **GLN 42** | **SER 496** |
| LYS 353 | **GLY 496** | **TYR 83** | **THR 500** | **TYR 83** | **ARG 498** |
| ASP 337 | **GLN 498** | **ASN 330** | **GLY 502** | **LYS 353** | **THR 500** |
| ARG 393 | **THR 500** | **LYS 353** | **VAL 503** |  | **TYR 501** |
|  | **ASN 501** | **ARG 393** | **TYR 505** |  | **GLY 502** |
|  | **TYR 505** |  |  |  | **HIE 505** |
