# Supplementary figures and images for: Different aspects in explaining how mutations could affect the binding mechanism of receptor binding domain of SARS-CoV-2 spike protein in interaction with ACE2
Source: PLoS One. 2023 Sep 8;18(9):e0291210. doi: 10.1371/journal.pone.0291210 (PMC10490914; doi:10.1371/journal.pone.0291210)

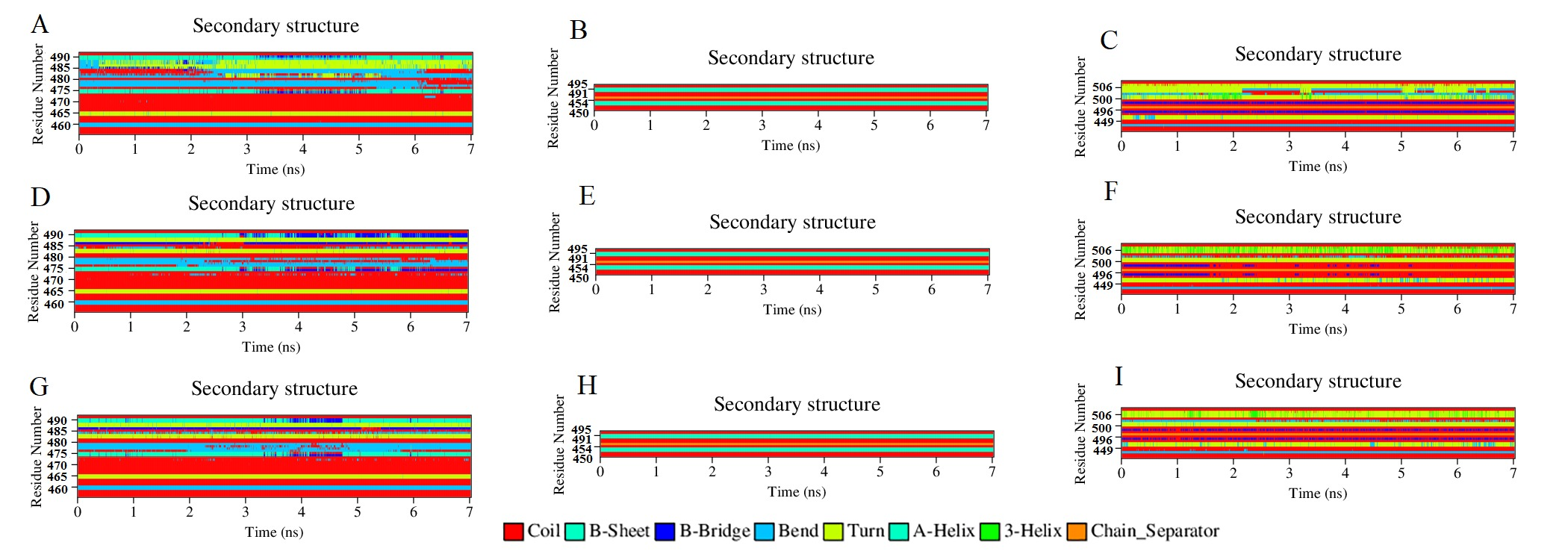

Supplement: S1 Fig — Changes of the secondary structure elements of CR1 domain (A, D, and G), CR2 domain (B, E, and H), and CR3 domain (C, F, and I) during SMD simulations. SARS-CoV-2 (A, B and C), Delta (D, E and F), and Omicron (G, H, and I). (TIF) [file pone.0291210.s003.tif]

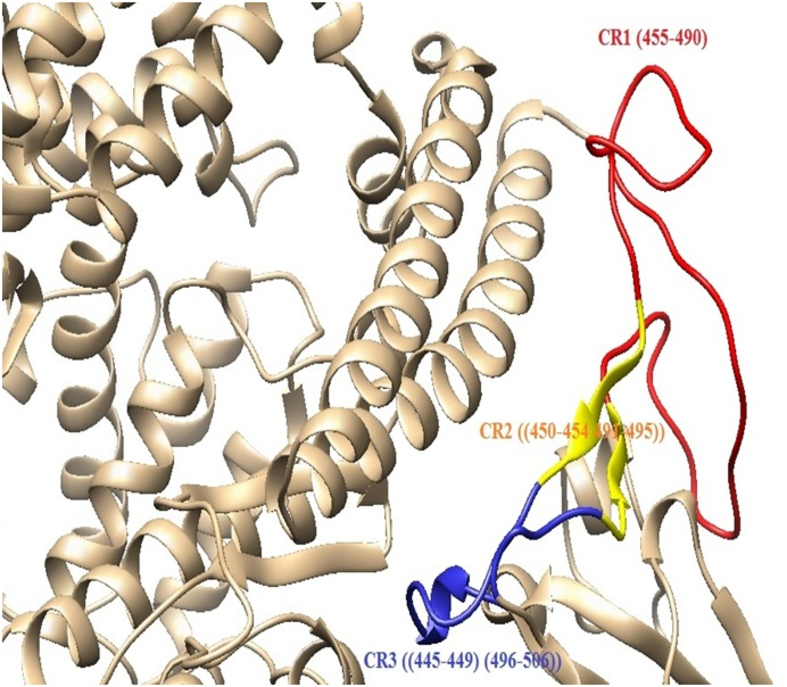

Supplement: S2 Fig — (TIF) [file pone.0291210.s004.tif]

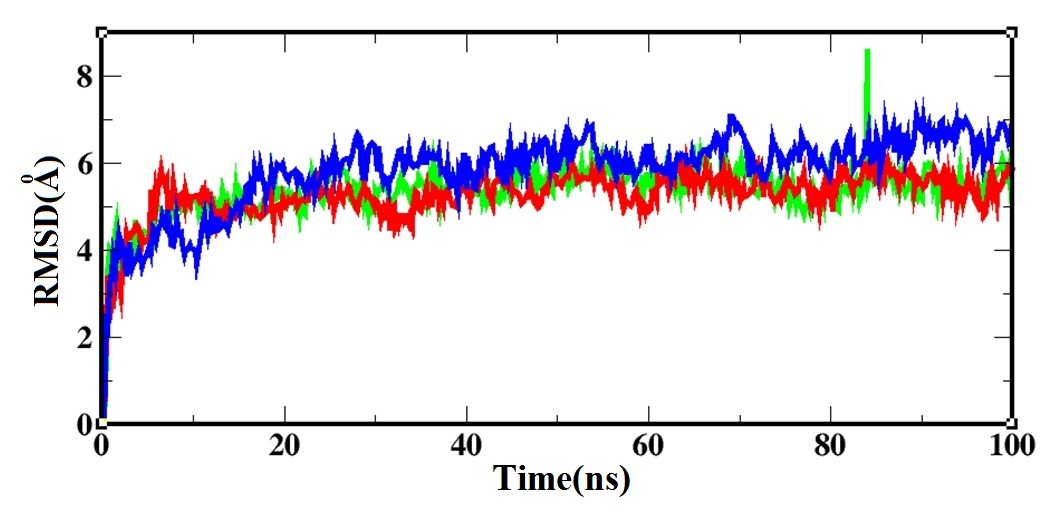

Supplement: S3 Fig — Wild Type- SARS-CoV2 (green color), Delta (red color), and Omicron (blue color). (TIF) [file pone.0291210.s005.tif]

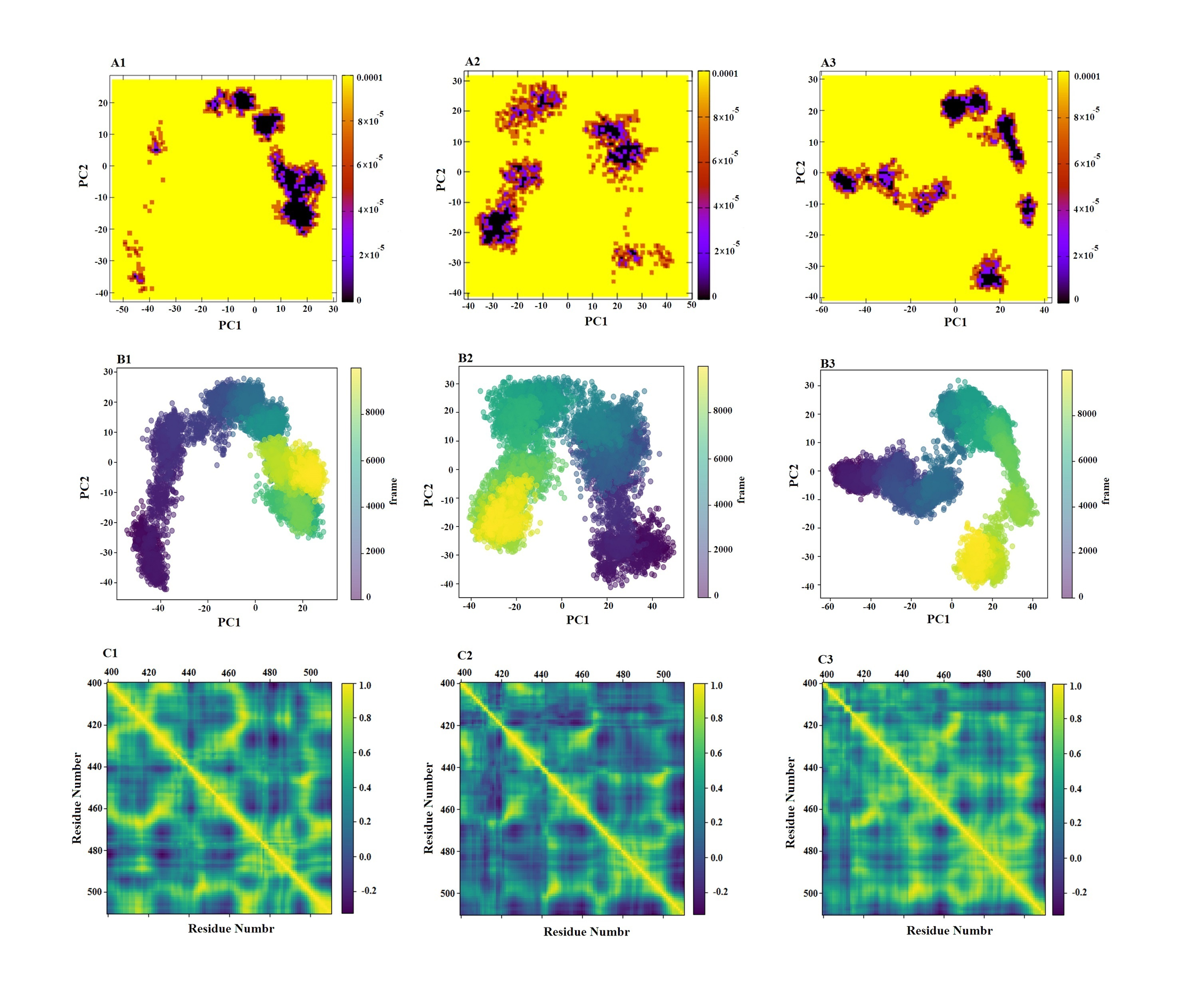

Supplement: S4 Fig — SARS-CoV-2 (A1, A2, A3), Delta (B1, B2, B3), and Omicron (C1, C2, C3) variants. (TIF) [file pone.0291210.s006.tif]
